# Bioluminescence imaging to track bacterial dissemination of *Yersinia pestis* using different routes of infection in mice

**DOI:** 10.1186/1471-2180-12-147

**Published:** 2012-07-24

**Authors:** Rodrigo J Gonzalez, Eric H Weening, Richard Frothingham, Gregory D Sempowski, Virginia L Miller

**Affiliations:** 1 Department of Microbiology and Immunology, University of North Carolina, Chapel Hill, NC, USA; 2 Department of Genetics, University of North Carolina, Chapel Hill, NC, USA; 3 Department of Medicine and Duke Human Vaccine Institute, Duke University Medical Center, Durham, NC, USA; 4 Department of Molecular Genetics and Microbiology, Duke University Medical Center, Durham, NC, USA; 5 Department of Pathology, Duke University Medical Center, Durham, NC, USA

**Keywords:** Plague, Bioluminescence, In vivo imaging, Bacterial dissemination

## Abstract

**Background:**

Plague is caused by *Yersinia pestis*, a bacterium that disseminates inside of the host at remarkably high rates. Plague bacilli disrupt normal immune responses in the host allowing for systematic spread that is fatal if left untreated. How *Y. pestis* disseminates from the site of infection to deeper tissues is unknown. Dissemination studies for plague are typically performed in mice by determining the bacterial burden in specific organs at various time points. To follow bacterial dissemination during plague infections in mice we tested the possibility of using bioluminescence imaging (BLI), an alternative non-invasive approach. Fully virulent *Y. pestis* was transformed with a plasmid containing the *luxCDABE* genes, making it able to produce light; this *lux*-expressing strain was used to infect mice by subcutaneous, intradermal or intranasal inoculation.

**Results:**

We successfully obtained images from infected animals and were able to follow bacterial dissemination over time for each of the three different routes of inoculation. We also compared the radiance signal from animals infected with a wild type strain and a Δ*caf1*Δ*psaA* mutant that we previously showed to be attenuated in colonization of the lymph node and systemic dissemination. Radiance signals from mice infected with the wild type strain were larger than values obtained from mice infected with the mutant strain (linear regression of normalized values, P < 0.05).

**Conclusions:**

We demonstrate that BLI is useful for monitoring dissemination from multiple inoculation sites, and for characterization of mutants with defects in colonization or dissemination.

## Background

*Yersinia pestis* is a highly virulent Gram-negative bacterial species that infects mammals and causes plague. Plague is a lethal disease known for its important role in history, mainly as the cause of the Black Death [1-3]. Due to the emergence of antibiotics [4], plague no longer poses the same threat to public health as it did in the past. However, the disease is still present in almost every continent [5] causing fatalities that, during the last two decades, have fluctuated between several hundred to several thousand deaths per year [6]. Plague is maintained in sylvatic animal reservoirs, and human populations that are in close contact with these reservoirs are at high risk [7]. Chemotherapy is efficacious only if administered early after infection and untreated individuals succumb to plague in less than a week. Furthermore, public health concerns have been raised because of reports of drug resistant strains in endemic foci [8].

The disease manifests after inhalation of bacteria suspended in aerosols (pneumonic plague) or through contact with broken skin (bubonic and septicemic plague) [9,10]. While pneumonic plague is the most virulent form of the disease, bubonic plague is the most prevalent perhaps due to its dynamics of transmission, for which a flea vector is essential [11]. Little is known about how *Y. pestis* disseminates within the host after infection. It is known, however, that at some point after infection, *Y. pestis* expresses a set of genes that impair host immune responses [12-14]. These factors are thought to be essential for bacterial dissemination. Dissemination during bubonic plague traditionally has been studied through experiments where different organs from infected mice are harvested at various time points post inoculation. Harvested organs are then homogenized and plated to obtain bacterial burden. These experiments have suggested that *Y. pestis* travels from the site of infection to draining lymph nodes (LN) prior to disseminating throughout the rest of the body [15,16]. Bacterial burden data from these experiments give a snapshot of a very narrow window (a specific organ at a specific time) through the course of infection. Furthermore, the approach is invasive, requires a large number of animals, and animals must be sacrificed at each time point making it impossible to keep track of the progression of infection on the same group of individuals.

*In vivo* bioluminescence imaging (BLI) is an approach that has been used to detect light-emitting cells inside of small mammals [17]. Using BLI, researchers have described and studied dissemination of viral, parasitic and bacterial pathogens within a host in a non-invasive manner [18-21]. Thus, the same group of animals can be imaged for as long as desired over the course of infection. The system requires that the pathogen produce luminescence, and infected animals are then imaged with a high-sensitivity camera that detects very small amounts of light. Non-luminescent bacteria can be genetically modified to express the *lux* genes (*luxCDABE*), which encode a bacterial luciferase and other enzymes that are necessary to generate substrate for luciferase [22]. In the presence of oxygen, luciferase catalyzes a reaction that produces light as a byproduct [23]. We transformed *Y. pestis* CO92 with plasmid pGEN-*luxCDABE* that contains the *luxCDABE* genes [24]. Using this strain of *Y. pestis* expressing the *lux* genes we determined that it is suitable for in vivo BLI after subcutaneous, intradermal and intranasal inoculation. In addition, we determined that BLI is suitable for the study of mutant strains that are attenuated or defective in dissemination or colonization during infection. This extends the findings of a recent report demonstrating the suitability of BLI to study *Y. pestis* infections by the subcutaneous route of inoculation [25].

BLI technology offers a new perspective to study the spread of *Y. pestis* in the host. This technology could be adopted in the future as an alternative to experiments that measured bacterial burdens in specific organs, facilitating the discovery and study of genes that are important in pathogenesis.

## Results

### The pGEN-*luxCDABE* vector is stable in *Y. pestis* during infection

**Figure 1:**
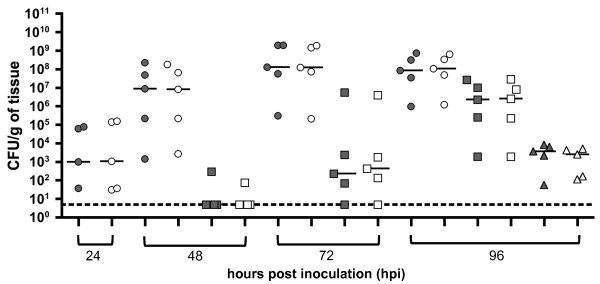
**Bacterial loads in C57Bl/6J mice infected subcutaneously with pGEN-*luxCDABE*-carrying*Y. pestis.*** Animals were infected with ~200 CFU at a cervical site. Organs were harvested and plated for bacterial counts at the indicated hours post inoculation on BHI alone (gray symbols) and BHI + carbenicillin (white symbols). Bacterial numbers are reported in CFU/g of tissue. Each mark represents a value from a single organ and the horizontal lines represent the median of the group. Superficial cervical lymph nodes are represented as circles, spleens as squares, and lungs as triangles. A dotted line represents the limit of detection. Data shown from a single experiment.

Bacteria carrying a reporter plasmid could potentially lose it at a specific site or time point during infection. A subpopulation lacking the plasmid could result in false negatives or decreases in signal detection that are not necessarily related to lower numbers of bacteria. To determine if pGEN-*luxCDABE* (pGEN-*lux*) was maintained during *Y. pestis* infections, we performed a kinetic study with mice infected with CO92 carrying pGEN-*lux*. Mice were inoculated subcutaneously (SC) and LN harvested at 24 hours post inoculation (hpi), LN and spleens harvested at 48 and 72 hpi, and LN, spleens and lungs harvested at 96 hpi. Homogenates of each organ were plated on BHI and BHI with carbenicillin. Bacterial enumeration showed no differences between the two growth conditions, indicating that pGEN-*lux* is stable in vivo up to 96 hpi in all organs tested (Figure 1). Additionally, organs from all animals imaged in this study were also plated on BHI and BHI with carbenicillin (after last imaging time point). We observed the same levels of plasmid stability that we report in Figure 1 (data not shown).

**Figure 2:**
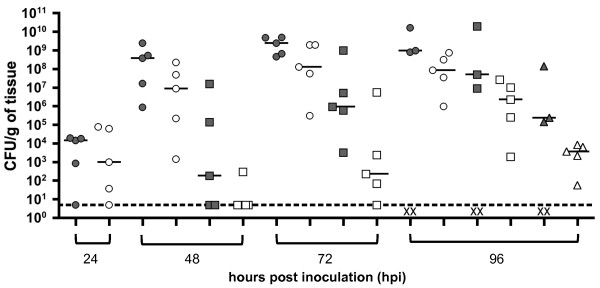
**Bacterial loads in C57Bl/6J mice infected subcutaneously with either wild type or pGEN-*luxCDABE*-carrying*Y. pestis.*** Animals were infected with ~200 CFU at a cervical site. Organs were harvested and plated for bacterial counts at the indicated hours post inoculation. Bacterial numbers are reported in CFU/g of tissue. Gray and white symbols represent organs from animals infected with *Y. pestis* and *Y. pestis* carrying pGEN-*luxCDABE*, respectively. Each mark represents a value from a single organ and the horizontal lines represent the median of the group. Superficial cervical lymph nodes are represented as circles, spleens as squares, and lungs as triangles. A dotted line represents the limit of detection and an x letter represents missing values of a specific tissue due to the death of an animal. Data shown from a single experiment.

Another important control experiment was to determine if pGEN-*lux* impacted the virulence of *Y. pestis*. Mice were inoculated with either *Y. pestis* alone or *Y. pestis* carrying pGEN-*lux*. Both groups of mice displayed signs of plague infection and mortality at similar times. However, the bacterial burden in tissues from mice infected with *Y. pestis* carrying pGEN-*lux* was lower in comparison to tissues from mice infected with *Y. pestis* without the plasmid (Figure 2). While bacterial counts suggest that pGEN-*lux* might cause a slight delay in the progression of infection, overt signs of plague were observed in all mice infected with either strain at comparable times. Additionally, all mice infected during our BLI experiments died at times expected from infections with a wild type strain. Since all strains used for BLI will carry the same plasmid, relative virulence attributes will be comparable despite the slight attenuation caused by pGEN-*lux*.

### BLI of *Y. pestis* after subcutaneous infection

**Figure 3:**
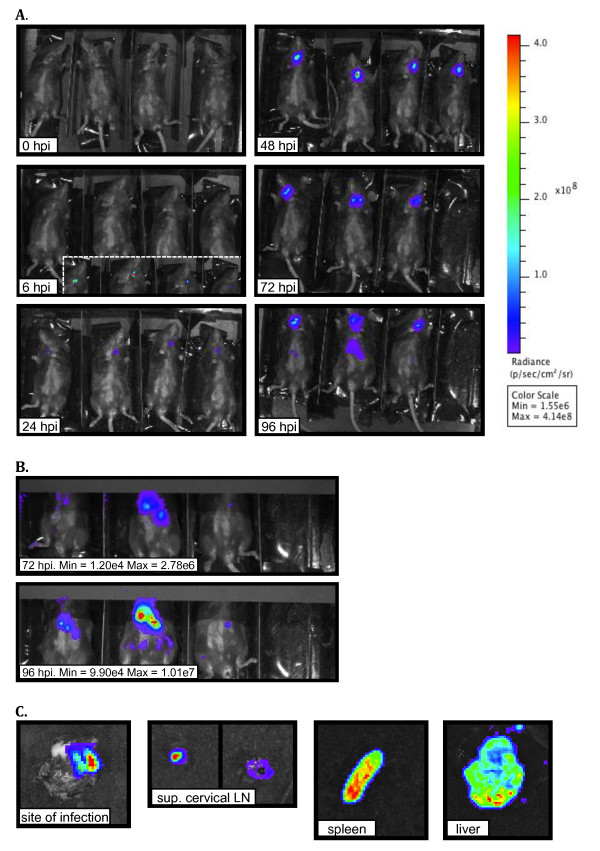
**BLI of C57BL/6J mice infected subcutaneously with Yp*lux***^***+***^**at a cervical site.** (**A**) Animals were inoculated with ~200 CFU and imaged at the indicated hours post inoculation (hpi). Luminescence signal is reported as radiance (p/sec/cm^2^/sr) in a scale paired with a color bar shown next to the images. For 6 hpi, the image in the window is shown using an individual color scale with radiance of Min = 8.53e3 and Max = 3.97e4. (**B**) Images of the abdomen at 72 and 96 hpi (same mice shown in panel A) under an individual radiance scale (Max and Min values are shown). (**C**) Site of inoculation [skin (inner side)], superficial cervical lymph nodes, spleen and liver (from one of the mice shown in A) imaged individually after dissection. Individual scales of radiance were used due to variability in signal (site of infection and liver, Min = 1.57e5 Max = 3.74e6; lymph nodes, Min = 2.10e6 Max = 2.28e8; spleen, Min = 1.73e5 Max = 1.38e7). Shown is a representative experiment.

**Figure 4:**
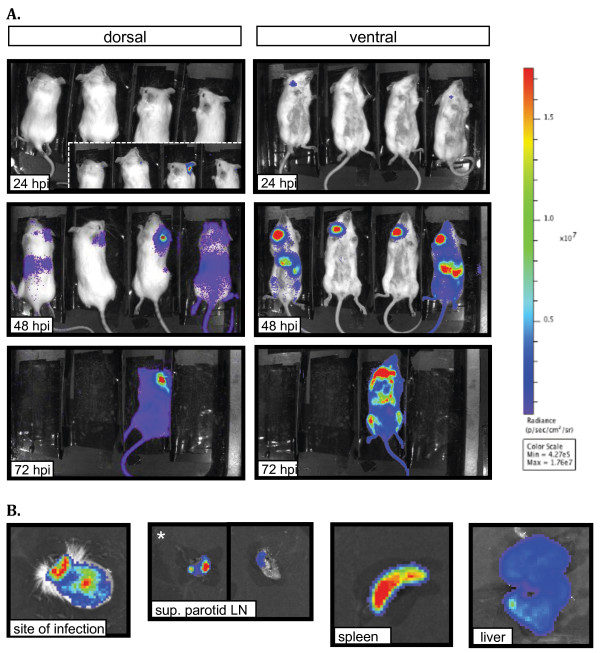
**BLI of B6(Cg)-*Tyrc-2J*/J mice infected intradermally with Yp*lux***^**+**^**in the ear pinna.** (**A**) Mice were inoculated with ~200 CFU and were imaged (ventral and dorsal sides) at the indicated hours post inoculation (hpi). Luminescence signal is reported as radiance (p/sec/cm^2^/sr) in a scale paired with a color bar shown next to the images. For 24 hpi (dorsal view), the window shows an image with signal at an individual radiance color scale with of Min = 1.11e4 and Max = 1.43e5. (**B**) Site of infection (right ear), superficial parotid right and left lymph nodes, spleen and liver (from one of the mice shown in A) imaged individually after dissection. An asterisk denotes the LN that drains the site of infection. Individual scales of radiance were used due to variability in signal (site of infection, Min = 1.89e4 Max = 8.97e4; lymph nodes, Min = 1.89e6 Max = 8.97e7; spleen and liver, Min = 5.25e5 Max = 2.34e7). Shown is a representative experiment.

In order to determine if BLI would be a suitable method for following dissemination or colonization of *Y. pestis* in vivo, we turned to the well-characterized subcutaneous model of infection [26]. C57BL/6J mice were inoculated SC with SC with Y. pestis CO92 transformed with the pGEN-*luxCDABE* plasmid (a strain we will refer to as Yp*lux*^+^ throughout the rest of this document), and the mice imaged at 0, 6, 24, 48, 72 and 96 hpi. Although the radiance levels were initially low, all animals had signal at the site of infection (neck) at 6 hpi, and the signal appeared to increase during the course of infection (Figure 3A). At 72 hpi, the region of radiance appeared to have two separate high intensity spots. The localization of these spots coincides with the approximate location of the superficial cervical LNs to which the site of infection is predicted to drain. Signal was also detected from the abdomen at 72 hpi. However, because of its low intensity, this signal is not evident in Figure 3A. All images in Figure 3A are standardized to the same radiance scale, thus low intensity spots are not visible. Low intensity spots, however, are visible when high intensity spots are covered. After covering high intensity spots from the neck with black opaque paper, we could visualize signal from the abdomen at 72 hpi (Figure 3B). Signal from the abdomen was not visualized before 72 hpi but quantification above background levels was obtained at 48 hpi (Figure 4C). At 96 hpi, radiance in the abdominal region increased in intensity (Figure 3A and B). From this and previous experiments, we observed that the presence and intensity of this signal tends to be variable among individuals. Also, from previous experiments where we imaged mice beyond 96 hpi, we determined that the presence of this signal, especially when high in intensity and spread in size, can be used as a predictor of death within the following 24 h. At time points subsequent to detection of light from the abdomen, signal was evident at sites where the skin was not covered by fur, such as the tail (data not shown). This might be the result of early stages of septicemia, where light from bacteria circulating in blood is only detectible from superficial vascularized tissue, such as the skin. At the latter stages of infection (>96 hpi), septicemia is evident as signal that can be detected from the entire animal.

Experiments in which bacterial load was measured showed that the LN are the first organs to be colonized, followed by deeper tissues (e.g. spleens and livers) [16]. The resolution provided by the BLI system, however, does not allow us to be certain that signal from the neck and abdomen comes from these organs. Therefore, mice were dissected to determine that signal indeed originated from LN, spleens and livers. These organs, along with the patch of skin where bacteria were inoculated, also were imaged individually at 96 hpi and found to emit light (Figure 3C). Thus, origin of light in specific organs is consistent with previous data measuring bacterial burden by plating macerated tissues.

### Dynamics of bacterial dissemination after intradermal infection in the ear pinna

Having established that BLI is a useful method to monitor dissemination following a SC infection, we wanted to determine the dynamics of dissemination of plague bacilli after intradermal (ID) infection. This model is rarely used for plague studies despite the fact that it may mimic a fleabite more closely than a SC inoculation [27]. We employed the ear pinna as the site of infection to guarantee that no subcutaneous tissue is reached [27]. In this model, the draining LN is the superficial parotid LN [as identified from [28]], which is distant from the site of infection. Thus, signal from the site of infection can be isolated from signal from the draining LN, a distinction not easily discerned in the SC model. Because the superficial parotid LNs are located deeper in the neck, we opted to infect B6(Cg)-*Tyrc-2J*/J mice. These mice differ from C57BL/6J in that pigment is absent from their skin. Using mice lacking skin pigments can increase light detection due to less absorption of light by the skin. Thus, the B6(Cg)-*Tyrc-2J*/J mice were a good alternative to maximize detection from small deeper tissues (i.e. superficial parotid LNs) without compromising our well characterized C57BL/6J model for bubonic plague.

**Figure 5:**
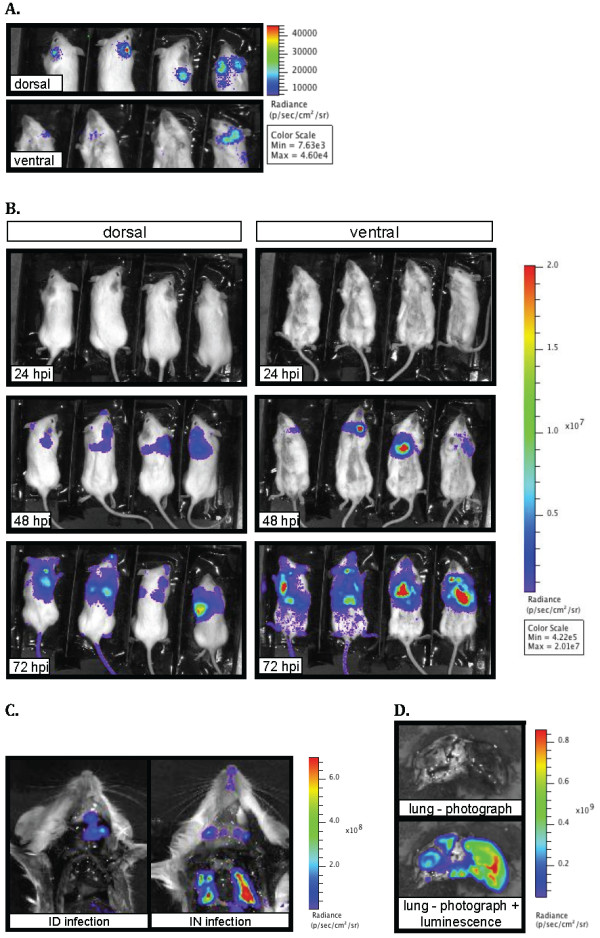
**BLI after Yp*lux***^**+**^**intranasal inoculation in the left nostril of B6(Cg)-*Tyrc-2J*/J mice.** (**A**) Mice were inoculated IN with ~10^4^ CFU.Images of the neck and head (dorsal and ventral) at 24 hpi under an individual radiance scale. The color bars serve as reference for radiance intensity (p/sec/cm^2^/sr; Min and Max values are shown) from each spot in the mouse from which signal was detected. (**B**) Images of the dorsal and ventral sides of the animals at different time points (shown in hpi). (**C**) Signal from the lungs after dissection in an animal infected ID in comparison to an animal infected IN (Min = 5.02e7 and Max = 8.62e8). (**D**) Isolated lungs showing a necrotic spot (photograph) and how highest levels of radiance (photograph + luminescence) originated from this spot (Min = 4.42e6 and Max = 7.02e8). Color bars serve as reference for radiance values. Shown is a representative experiment

The ear pinna was inoculated with ~200 CFU and animals were imaged at different time points (Figure 5A). Low levels of signal from the site of infection could be detected in some animals at 6 hpi (data not shown). However, at 24 hpi, strong signal was consistently detected in the ear. In addition, some of the mice had detectible signal in the right side of the neck, approximately where the superficial parotid LN is located. At 48 hpi light signal from the site of infection appeared to increase considerably. At this same time point, signal from the parotid LN increased dramatically, and light was detected in the abdomen and rest of the body in some animals, indicating systemic dissemination. At 72 hpi only one mouse had survived and it showed high levels of signal from the whole body, indicating advanced stages of septicemic dissemination. The right superficial parotid LN was confirmed as the highest source of radiance from the neck after dissection of this mouse (Figure 5B). As previously reported for latter stages of infection [16], the LN that drains the site of infection was not the only LN that appeared to be colonized. However, the superficial parotid LN that drains the site of infection (white asterisk, Figure 5B) appeared to emit higher levels of radiance in comparison to other LNs. Isolated spleens and livers were imaged to confirm them as the source of signal from the abdominal area(Figure 5B).

### Bacterial dissemination during pneumonic plague

Pneumonic plague is less common but more fulminant than bubonic plague, and is the only form of the disease that can be transmitted directly from human to human (does not require a flea vector). We used BLI to follow dissemination of *Y. pestis* after intranasal inoculation*,* a well-characterized model for pneumonic plague [29]. Lung tissue is the primary tissue colonized by *Y. pestis* during pneumonic infections. Because the lungs reside in the thoracic cavity covered by other organs and bone, we again used B6(Cg)-*Tyrc-2J*/J mice to increase the probability of detecting signal from lung tissue.

In some isolated cases, radiance was detected from the abdomen and from feces at 6 hpi (data not shown). This signal was not detected at any latter time points and presence of abdominal or fecal signal did not appear to alter the course of infection in the animals where it was detected.

**Figure 6:**
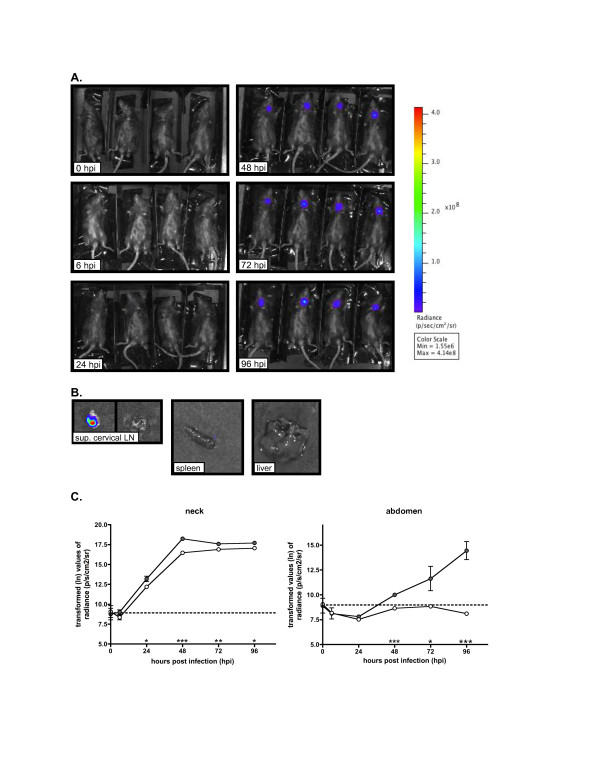
**BLI of C57BL/6J mice infected subcutaneously with Δ*caf1*Δ*psaA Y. pestis*carrying the pGEN-*luxCDABE*vector.** (**A**) Mice were inoculated with ~200 CFU of the double mutant. Images correspond to infected animals at different time points post inoculation (shown in hpi). A color bar serves as a reference for the radiance scale (p/sec/cm^2^/sr) used to standardize all images. (**B**) Images of superficial cervical lymph nodes, spleen and liver (from one of the mice shown in A) imaged individually after dissection. Luminescence was detected only from lymph nodes, imaged in an individual scale of radiance with a Min = 2.28e6 and Max = 4.27e7. (**C**) Transformed values (ln) of the mean radiance per group from the neck (left) and abdomen (right) from animals infected with Yp*lux*^*+*^ (gray circles) and YpΔ*caf1*Δ*psaA lux*^+^ (white circles)*,* as determined by measurements from regions of interest (ROI) of images from two independent experiments. A dotted line depicts background radiance levels. Asterisks denote statistical significance in differences between the two means compared in a time point, as determined by linear regression analysis of transformed values (*, P < 0.05; **, P < 0.005; ***, P < 0.0005). Error bars represent the standard error of the mean (SEM). Shown is a representative experiment

Very little light was detected in the mice at 24 hpi, at which time some mice showed signal from different regions in the neck or on the head (Figure 6A). At 48 hpi, light was detected in all animals, mainly from the mid and upper thorax (Figure 6B). Radiance spread and intensity increased considerably at 72 hpi, a time at which all mice showed pronounced signs of disease. Immediately after imaging at 72 hpi, one of the four mice in the group was sacrificed and dissected to determine the source of light. The lungs were determined to be the source of luminosity from the thorax, and light from this organ was confirmed to be unique to IN infections as animals infected using other routes (e.g. ID, Figure 6C) did not show signal from the lungs. Additionally, we observed that IN-inoculated animals showed signal from the tip of the nose (visible in Figure 6C) indicating that bacteria were present at the site of inoculation at 72 hpi. Upon dissection of the lungs, we noticed that part of the organ was necrotic in appearance; imaging of isolated lungs showed that the necrotized tissue produced higher levels of signal (Figure 6D) in comparison to other areas of the lung. While Figure 6C and 6D show data from only one mouse, we performed this experiment multiple times and in all cases we made the same observations mentioned above (data not shown).

### BLI to identify mutants with defects in dissemination or colonization

One of the goals of this study was to determine whether mutants with a defect in colonization and/or dissemination could be identified by BLI. As proof of concept, we compared radiance from mice infected with Yp*lux*^+^ or YpΔ*caf1*Δ*psaAlux*^+^ mutant. Caf1 and PsaA previously were shown to play a role in dissemination and colonization in an additive manner [30]. The SC model of infection and C57BL/6J mice were chosen for this comparison because the colonization phenotype of the Δ*caf1*Δ*psaA* strain was originally tested using this model. BLI revealed that the Δ*caf1*Δ*psaA* strain was attenuated in dissemination or colonization to deeper tissues from the LN, in agreement with previous work [30] (Figure 4A and B)*.* Radiance measurements allowed us to determine that signal intensity in the neck was lower in animals infected with the double mutant strain in comparison to those infected with Yp*lux*^+^, indicating that colonization of the LN by the Δ*caf1*Δ*psaAlux*^+^ mutant also was impaired compared to wild type, in agreement with previous work [30] (Figure 4C). Differences of radiance values from mice infected with Yp*lux*^+^ against Δ*caf1*Δ*psaAlux*^*+*^ attained statistical significance at 24, 48, 72 and 96 hpi (linear regression analysis of normalized values, P < 0.05).

Mice infected with the Δ*caf1*Δ*psaA* strain never displayed detectible signal from the abdomen at any time point (Figure 4A). The radiance values from the abdomen of these mice were below background levels at each time point examined. These radiance values were subjected to regression analysis and determined to be significantly different from the values obtained from mice infected with Yp*lux*^+^ at 48, 72 and 96 hpi. To determine if the absence of signal in YpΔ*caf1*Δ*psaAlux*^+^-infected mice was due to extremely low levels that were blocked by skin or other tissue, we dissected the mice and imaged isolated spleens and livers at 96 hpi. No signal was detected from the individual organs (Figure 4B). In addition, all animals infected with the Δ*caf1*Δ*psaA* mutant survived past 96 hpi and never showed any signs of disease. We continued to image these animals up to 168 hpi, and found that the signal from the neck never disappeared and that bacteria appeared to be contained at this site (data not shown). Overall, imaging from mice infected with YpΔ*caf1*Δ*psaAlux*^+^confirmed previous findings in C57BL/6J where bacteria were detected in LN, but at lower numbers in comparison to mice infected with a wild type strain, and never or rarely were detected in spleens [30]*.*

## Discussion

Plague is a disease with devastating effects on the host that are fatal if left untreated. These effects are the result of the ability that *Y. pestis* displays to suppress host immune responses and to promote systemic dissemination at remarkably high rates. Numerous studies have described many virulence factors that are essential to suppress host immune responses [2,31]. The direct contributions of these virulence factors to bacterial dissemination, however, are still unclear. The study of dissemination per se is a field that is lagging behind in plague research. BLI is a tool that allows for the visualization of a pathogen in a host during infection and a very promising alternative to better understand *Y. pestis* dissemination. A recent report described the use of BLI in a subcutaneous (SC) model of bubonic plague [25]. In this report, the pGEN-*luxCDABE* plasmid was described to have no effect on the virulence of *Y. pestis* and to be suitable for BLI as luminosity correlated with bacterial counts in vivo; our results confirmed and expanded upon these findings. Our goal was to determine whether BLI could be used to follow dissemination and colonization of *Y. pestis* in mice after using different routes of inoculation that closely mimic bubonic and pneumonic plague. Moreover, we tested whether BLI could be used to detect mutants with defects in colonization or dissemination.

After inoculation with a strain of *Y. pestis* that contains pGEN-*luxCDABE*, we showed that animals can be imaged through the course of infection in such a way that bacterial spread could be followed over time for three different models of infection. Our results from the SC inoculation model support the previous notion that, during bubonic plague, *Y. pestis* travels from the site of inoculation to the proximal lymph node prior to dissemination to deeper tissues [16]. We observed that bacteria were maintained at the site of inoculation during the course of infection, as previously reported for ear intradermal (ID) infections [15]. For both, the SC and ID models, the bacterial population at the site of inoculation appeared not only to be maintained, but also to expand. However, while we quantified signal from the site of infection in the SC-inoculated animals, we cannot conclude such signal comes from the skin alone. In our SC model, the patch of inoculated skin is located in an anatomical position on top of the superficial cervical LNs and thus, both, skin and LNs, are imaged as a single source of radiance. We could determine that signal was coming partly from the site of inoculation after removing the patch of skin and imaging it individually. This complication is minimized in the ID model, where the site of inoculation (ear pinna) is distant from the draining LN (superficial parotid LN). While an increase overtime in signal intensity from the ear was observed, we were not able to quantify the signal, as it was difficult to place the ears of all mice at the same position inside of the animal isolation chamber.

Images taken during the first hours following intranasal (IN) infections suggested that, in isolated cases, at least part of the inoculum can go to the stomach. The IN route requires delivering small drops of inoculum into one of the nostrils (total volume of 20 μL), and some of this inoculum could be swallowed rather than inhaled. Signal from the stomach never seemed to last beyond the 6 hpi time point, suggesting that gastric infections with *Y. pestis* in these mice are cleared quickly. We also observed that the feces of half of the mice produced detectible signal, indicating that *Y. pestis* was being shed. This was only observed at very early time points (6 hpi), indicating that bacteria were fully shed from the gastrointestinal tract by 24 hpi. In humans, it has been shown that transmission can occur after ingestion of contaminated food [32]. While mice are coprophagous, it is not know whether a fecal-oral route could be a mechanism for *Y. pestis* to disperse or infect other individuals. Detecting signal from the tip of the nose also opens the question whether bacteria could be transmitted to other individuals with whom food and water are shared. We do not know whether signal from the stomach or the tip of the nose would still be present after an aerosol infection, a route that pneumonic plague is assumed to be transmitted in nature. All mice, independent of the presence of signal from the stomach or feces, showed the same progression of infection with comparable levels of signal from the thorax. More importantly, all animals showed signs of disease and mortality at very similar times. This observation suggests that the fraction of the inoculum that may go to the gastrointestinal tract has no effect on the overall pneumonic infection.

The low number of mice used during BLI is one of its more important advantages. However, it can also be a disadvantage because of the variability in bacterial load for a specific organ from animal to animal and sudden death, both inherent aspects of plague infections. The differences in the levels of significance from time point to time point when comparing radiance values between the wild type and double mutant infected animals are due to this high variability of bacterial load and death. Despite these challenges, we found that BLI is a suitable method for studying dissemination/colonization of *Y. pestis* in three separate models of plague, and that significant differences in radiance could be detected between wild type and a mutant of modest attenuation using relatively few mice.

## Conclusions

We used BLI to follow bacterial dissemination in mice after SC, ID and IN infections. The dissemination patterns we describe are fully consistent with dissemination and colonization data that has been reported for bubonic and pneumonic plague experiments that describe bacterial burden in specific organs after infection. In addition, we found lower levels of signal from a mutant with established defects in colonization and dissemination in comparison to a wild type strain, indicating that this will be a useful technique for mutational analysis. We believe that BLI is a powerful alternative, and complement, to the approaches that are currently used for plague dissemination studies.

## Methods

### Bacterial strains and cultures

*Y. pestis* CO92 and *Y. pestis* CO92 Δ*caf1*Δ*psaA* were transformed with pGEN-*luxCDABE *[24]. This plasmid contains the Hok/Sok toxin/antitoxin system enabling plasmid maintenance in vivo without antibiotic selection. Throughout this document we referred to *Y. pestis* CO92 transformed with the pGEN-*luxCDABE* plasmid as Yp*lux*^+^, to *Y. pestis* CO92 Δ*caf1*Δ*psaA* transformed with the same plasmid as YpΔ*caf1*Δ*psaAlux*^+^ or simply as “double mutant” and to the pGEN-*luxCDABE* plasmid itself as pGEN-*lux*. Bacteria transformed with pGEN-*lux* were cultured in the presence of carbenicillin at 100 μg/mL, unless BHI alone is stated as growth medium. Bacteria were plated on brain heart infusion (BHI) agar (BD Biosciences, Bedford, MA) plates and incubated for 48 h at 26°C. For intranasal inoculations, liquid cultures were incubated at 37°C in the presence of 2.5 mM CaCl_2_ as previously described [29]. For subcutaneous and intradermal inoculations, liquid cultures were incubated at 26°C for 15 h. All strains (Yp*lux*^+^, YpΔ*caf1*Δ*psaAlux*^+^ and *Y. pestis* lacking pGEN-*lux*) showed comparable optical density (OD_600_) values after culturing in liquid broth. To obtain the final inocula for all infections, liquid cultures were serial diluted in phosphate buffered saline (PBS). All procedures involving *Y. pestis* were conducted under strict biosafety level three conditions.

### Animal infections and tissues

Five-to-ten-week old female C57BL/6J or B6(Cg)-*Tyrc-2J*/J mice (Jackson Laboratory, Bar Harbor, ME) were subjected to subcutaneous (SC), intranasal (IN) or intradermal (ID) inoculation after providing anesthesia (2% isoflurane for SC and ketamine/xylazine for IN and ID). For SC inoculations, a volume of 100 μL was injected in the subcutaneous space at an anterior cervical site. The ear pinna was injected with a volume of 10 μL for ID inoculations. A volume of 20 μL was delivered into the left nostril of the animal for IN inoculations. The inoculum for the SC and ID inoculations was ~200 CFU, and ~10^4^ CFU for the IN inoculation.

For the determination of plasmid stability and strain characterization experiments, superficial cervical lymph nodes, spleens and lungs were removed from SC-infected mice after sacrificing the animals by injection of sodium pentobarbital. Plasmid stability was assessed by comparing bacterial counts after plating on BHI alone and BHI with carbenicillin. Strain characterization was determined by comparing bacterial counts of Yp*lux*^+^ against *Y. pestis* lacking the plasmid.

All procedures involving animals were approved by the University of North Carolina and Duke University Animal Care and Use Committees, protocols 11–128 and A185-11-07, respectively.

### *In vivo* Imaging

To enhance signal detection, the fur was shaved from the ventral and cervical regions of the mice with an electric razor two days before inoculation. Animals were anesthetized with 2% isoflurane during the entire imaging process, except for the time point 0 h post inoculation (hpi) for IN and ID, where the animals were still under the sedation from the ketamine/xylazine treatment. Prior to imaging, mice were placed in an animal isolation chamber (Caliper) to maintain containment of *Y. pestis* outside the biosafety cabinet. We used four mice per group, as this is the maximum number of mice that can be placed in the isolation chamber to be imaged at one time. Mice were imaged with an IVIS Spectrum instrument (Caliper) at 0, 6, 24, 48, 72 and 96 hpi, unless animals died or had to be sacrificed because of advanced signs of plague. The same group of mice was imaged at each time point. Every image was taken after placing the mice in the isolation chamber in the same order relative to one another. After imaging the last time point, mice were sacrificed with an overdose of isoflurane and one animal per group was dissected. The dissected individual was imaged to identify luminescence from specific organs. Organs were then removed from the animal and imaged individually to confirm the origin of signal. The remaining animals were sacrificed and their organs (LN, spleens or lungs) were removed, macerated and plated to compare bacterial load with previous reports for each model and to confirm plasmid stability as described above. Radiance signal was measured in photons/sec/cm^2^/steradian and analyzed using Living Image Software V.4.2 (Caliper). Radiance signal from a specific site (site of inoculation or abdomen) was quantified by defining a region of interest (ROI), which was drawn and measured using the Living Image Software (Caliper). Radiance background levels were obtained by measuring radiance from a ROI (from either site of inoculation or abdomen) of all animals imaged at 0 hours after inoculation. When signal was detected from one site (e.g. the neck) and not from a second site (e.g. the abdomen), the light emitting site from which signal was detected was covered with black opaque paper to increase image sensitivity. A specific site was considered to be negative (lacking signal) if no signal was observed after covering all other irradiating sites or if quantification of signal was below background levels. Radiance values from each ROI were transformed into log values to normalize their distribution. Linear regression analysis of these values was performed in STATA 12 (Stata Corp, College Station, TX) to test differences in average radiance between groups. A two sided P value <0.05 was set to determine statistical significance.

## Competing interests

The authors declare that they have no competing interests.

## Authors’ contributions

RJG was essential during all the experiments described in the study, participated in the experimental design, analyzed the data and drafted the manuscript. EHW was essential during the imaging experiments, participated in the experimental design and helped with critically revising the manuscript. RF contributed to experimental design and revision of the manuscript. GDS contributed to experimental design and revision of the manuscript. VLM participated in the coordination and design of the study and revised the manuscript for intellectual content. All authors read and approved the manuscript.
